# Evaluating quality of care at the end of life and setting best practice performance standards: a population-based observational study using linked routinely collected administrative databases

**DOI:** 10.1186/s12904-022-00927-2

**Published:** 2022-04-12

**Authors:** Mariska G. Oosterveld-Vlug, Marianne J. Heins, Manon S. A. Boddaert, Yvonne Engels, Agnes van der Heide, Bregje D. Onwuteaka-Philipsen, Anna K. L. Reyners, Anneke L. Francke

**Affiliations:** 1 Netherlands Institute for Health Services Research (Nivel), Utrecht, the Netherlands; 2 Netherlands Comprehensive Cancer Organisation (IKNL), Utrecht, the Netherlands; 3 Department of Anesthesiology, Pain and Palliative Medicine, Radboud University Medical Center, Nijmegen, the Netherlands; 4 Department of Public Health, Erasmus MC, University Medical Center Rotterdam, Rotterdam, the Netherlands; 5 Department of Public and Occupational Health, Amsterdam Public Health Research Institute, Amsterdam UMC, Vrije Universiteit Amsterdam, Amsterdam, The Netherlands; 6 Department of Medical Oncology, Center of Expertise in Palliative Care, University Medical Centre Groningen, University of Groningen, Groningen, the Netherlands

**Keywords:** End-of-life care, Quality indicators, Routinely collected health data, Benchmarking, Performance standards, Place of death, Hospital care

## Abstract

**Background:**

A high percentage of people dying at home, and a low percentage of people being admitted to hospital and dying there are regarded as indicators of appropriate care at the end of life. However, performance standards for these quality indicators are often lacking, which makes it difficult to state whether an indicator score falls between the ranges of good or poor quality care. The aim of this study was to assess quality indicators concerning place of death and hospital care utilization in people with diseases relevant for palliative care, and to establish best practice performance standards based on indicator scores in 31 regions in the Netherlands.

**Methods:**

A retrospective nationwide population-based observational study was conducted, using routinely collected administrative data concerning persons who died in 2017 in the Netherlands with underlying causes relevant for palliative care (*N* = 109,707). Data from four registries were linked for analysis. Scores on eight quality indicators concerning place of death and hospital care utilization were calculated, and compared across 31 healthcare insurance regions to establish relative benchmarks.

**Results:**

On average, 36.4% of the study population died at home (range between regions 30.5%-42.6%) and 20.4% in hospital (range 16.6%-25.5%). Roughly half of the population who received hospital care at any time in the last year of life were found to (also) receive hospital care in the last month of life. In the last month, 32.0% of the study population were admitted to hospital (range 29.4-36.4%), 5.3% to an Intensive Care Unit (range 3.2-6.9%) and 23.9% visited an Emergency Department (range 21.0-27.4%). In the same time period, less than 1% of the study population was resuscitated in hospital or received tube or intravenous feeding in hospital.

**Conclusions:**

The variation between regions points towards opportunities for practice improvement. The best practice performance standards as set in this study serve as ambitious but attainable targets for those regions that currently do not meet the standards. Policymakers, healthcare providers and researchers can use the suggested performance standards to further analyze causes of variance between regions and develop and test interventions that can improve practice.

## Introduction

At the end of life, the majority of people prefer to remain at home until death [1–4]. Hospital admissions at the end of life are often regarded as undesirable, particularly when they have an acute character [5–7]. Common reasons for hospitalization at the end of life are shortness of breath, pain, digestive or cardiovascular symptoms, delirium or loss of consciousness [6]. However, these symptoms can often be relieved at home [6–9]. Research in the Netherlands and other western countries shows that, in retrospect, 7–33% of the hospitalizations in the last three months of life of patients who died non-suddenly could have been avoided [7–10].

In order to improve care at the end of life and decrease the number of avoidable hospitalizations, insight into actual hospital care utilization and quality of care is an important first step. If available, these data can best be derived from existing registries, in order to prevent additional registration or measurement burden for patients and healthcare professionals. Quality of care can be assessed by using quality indicators. A few years ago, a group of experts in palliative care developed a set of quality indicators that measure aspects of care that may indicate potentially appropriate or inappropriate care at the end of life in people who died with dementia, cancer or COPD [11]. After all, medical care which is justifiable for patients with good prognosis can turn into inappropriate care near the end of life, as benefits of care no longer outweigh the possible negative effects of continuing this care. The set of quality indicators was developed specifically to be used at a population level, using administrative healthcare data.

However, quality indicators regarding care at the end of life often lack broadly accepted performance standards, which makes it difficult to state whether an indicator score points to high or poor quality of care. When performance standards are lacking, benchmarking can be done in order to formulate attainable targets indicating high-quality care [12]. In benchmark studies, scores on quality indicators are being compared between care organizations, regions or countries. Benchmarking allows the determination of relative ‘best practice’ performance standards rather than static absolute performance standards, and subsequently helps to identify the areas where the gap between one’s score and that of the best performers is the largest. As such, analyzing variation in quality indicators may lead to opportunities for practice improvements where needed. Previous population-level research in Belgium found substantial variation in healthcare use across regions in Belgium [13]. Such research has not been performed in the Netherlands yet.

Therefore, the aims of the presented study were threefold:

- (1) To gain insight in place of death and hospital care utilization at the end of life for people with diseases relevant for palliative care in the Netherlands;
- (2) To assess and compare scores on quality indicators concerning place of death and hospital care utilization at the end of life between 31 healthcare insurance regions in the Netherlands; and.
- (3) To develop best practice performance standards based on this comparison.

## Methods

### Study design and data sources

A retrospective nationwide population-based observational study was conducted, using routinely collected administrative data concerning persons who died in 2017 in the Netherlands. Administrative data from the following registries were linked for analysis:

- Three nationwide registries available at Statistics Netherlands: (1) Death certificate registry containing all reported deaths within a specific year, including date of death, cause of death and place of death (2017); (2) Sociodemographic registry containing information on gender, year of birth, ethnitcity and other characteristics of individuals residing in the Netherlands (up to 2017); (3) Registry containing postal code areas belonging to the place of residence of individuals residing in the Netherlands (up to 2017).
- The National Basic Registration Hospital Care (2016–2017) of Dutch Hospital Data, containing nationwide data of hospital admissions in all general and academic hospitals in the Netherlands.

All registries were linked in the secure environment of Statistics Netherlands, guaranteeing anonymity of the deceased.

### Study population

People with a wide range of chronic conditions, who face a trajectory of serious and life-limiting illness, may benefit from palliative care at the end of life. We therefore identified all decedents in 2017 who were potentially in need of palliative care, as based on a set of relevant underlying causes of death (ICD-10 codes) described by Etkind et al. (2017) [14] (Table 1).

**Table 1:** Causes of death (ICD-10 codes) indicating a potential need for palliative care (Etkind et al., 2017)

**Cause of death**	**ICD-10 codes**
Cancer	C00-C97
Heart disease and heart failure	I00-I52 (excl. I12 and I13)
Chronic lower respiratory disease, respiratory failure	J40-J47, J96
Haemorrhagic, ischaemic and unspecified stroke	I60-I69
Reno-vascular disease, renal failure	I12, I13, N17, N18, N28
Liver disease	K70-K77
Dementia, vascular dementia, Alzheimer’s disease, senility	F01, F03, G30, R54
Neurodegenerative disease (Huntington’s disease, motor neurone disease, Parkinson’s disease, progressive supranuclear palsy, multiple sclerosis, multi system atrophy)	G10, G12.2, G20, G23.1, G35, G90.3
HIV	B20-B24

Based on the postal code areas of their residence, persons dying from diseases relevant for palliative care could be divided over 31 regions of healthcare insurance offices (in Dutch ‘zorgkantoren’) that were operational in the Netherlands in 2017. These offices are linked to the healthcare insurer with the largest number of insured clients in a specific region. Healthcare insurance offices are responsible for ensuring high quality long-term care for clients in their region. In this regard, they make contracts with GPs, home care organizations and long-term care facilities (LTCFs) (i.e. nursing homes and residential care homes), and define requirements for delivering high-quality care. With regard to care at the end of life, healthcare insurance offices can for instance require that healthcare providers follow the standards of the national Quality Framework on Palliative Care [15].

### Measures

Place of death and hospital care utilization were assessed by using quality indicators from a previously developed and validated set [11]. Based on the availability of data within the registries and their relevance to a broad spectrum of diseases relevant for palliative care, eight quality indicators were selected from this set (Table 2). Except for the indicators regarding place of death, we assessed all indicators in various time periods before death, i.e. 360 days, 180 days, 90 days, 30 days, and 7 days before death.

**Table 2:** Selected quality indicators

**Quality indicator**	**Indicator of appropriate (A) or inappropriate (I) care**
**Place of death**	
% of people who died at home	A
% of people who died in hospital	I
**Hospital admissions and visits**	
% of people with ≥ 1 hospital admission	I
% of people with ≥ 1 acute^a^ hospital admission	I
% of people with ≥ 1 Intensive Care Unit (ICU) admission	I
% of people with ≥ 1 Emergency Department (ED) visit	I
**Treatments in hospital**	
% of people who were resuscitated in hospital	I
% of people who received tube feeding or intravenous feeding in hospital	I

^a^ An acute hospital admission is an admission that can not be delayed because immediate treatment or care is needed within 24 h according to the medical specialist

### Statistical analyses

The characteristics of the study population, their place of death and hospital care utilization in different time periods before death (study aim 1) were calculated using descriptive statistics. To assess and compare the scores on quality indicators between healthcare insurance regions (study aim 2), we used the period of 30 days before death, as most quality indicators are validated for this time period. To obtain a fair comparison across the 31 healthcare insurance regions, we matched each healthcare insurance region sample with the total population from which it was drawn on the demographic variables that were available in our dataset. We corrected for population differences in age (in 4 categories < 18, 18–64, 65–84, 85 or older), sex (male, female), cultural background (western, non-western) and cause of death (9 categories, as defined in Table 1) by applying a weighting method known as raking. With raking, each individual in a particular healthcare insurance region receives a weight as to resemble the characteristics of the total study population. We accounted for these weights when we assessed the scores on the quality indicators for each healthcare insurance region. To establish best practice performance standards (study aim 3), we followed a method that other researchers used before [13], and calculated quartiles of scores for each quality indicator across healthcare insurance regions. For the quality indicator ‘percentage of people dying at home’, associated with appropriate end-of-life care, the best practice performance standard was established at the upper margin of the third quartile or above. For all other indicators, associated with inappropriate care, the performance standard was established at the upper margin of the first quartile or below.

All analyses were conducted with SPSS version 25.

### Ethics

According to Dutch law, no ethical approval was required for this study, since posthumous collection of patient data is allowed in the Netherlands [16].

## Results

### Population characteristics

In total, 150,214 persons died in the Netherlands in 2017, of whom 109,707 persons (73%) died from diseases relevant for palliative care. Of these 109,707 persons, the majority died from cancer (40.9%), heart disease (23.6%) or dementia (15.6%) (Table 3). Demographic characteristics varied between disease groups. Persons dying from dementia were predominantly female (68.2%), of older age when they died (mean 87.3 years) and very few had a non-western cultural background (1.5%). On the other end, persons dying from liver disease were predominantly male (63.2%), of a younger age when they died (mean 67.1 years) and a bigger proportion had a non-western background (7.0%).

**Table 3:** Characteristics of the population dying from diseases relevant for palliative care

**Cause of death**	**N decedents (%)**	**% female**	**Age, mean (SD)**	**% with non-western cultural background**^**a**^
Cancer	44,908 (40.9)	45.3	73.0 (12.4)	3.3
Heart disease	25,840 (23.6)	51.2	81.5 (11.8)	3.2
Dementia	17,148 (15.6)	68.2	87.3 (7.2)	1.5
Stroke	9,199 (8.4)	59.0	81.9 (11.0)	3.2
Chronic respiratory disease	7,129 (6.5)	50.0	78.4 (10.4)	2.0
Neurodegenerative disease	2,776 (2.5)	44.5	77.5 (10.9)	2.5
Reno-vascular disease	1,578 (1.4)	54.1	83.3 (9.9)	5.0
Liver disease	1,104 (1.0)	36.8	67.1 (13.4)	7.0
HIV	25 (0.0)	-	-	-
Total	109,707 (100)	51.8^b^	78.5 (12.5)^b^	2.9^b^

^a^ Persons with a Turkish, Moroccan, Surinam, Antillian or other non-western background

^b^ To guarantee anonimity, the characteristics of the persons within the small group of persons with *HIV* were not included in this table

### Place of death

**Fig. 1:**
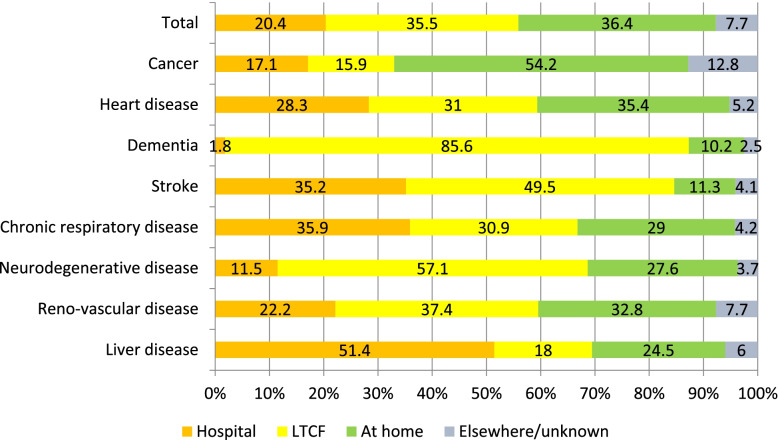
Place of death, by cause of death (*N* = 109,682 [To guarantee anonimity, the characteristics of the persons within the small group of persons with HIV were not included in this figure])

In 2017, 20.4% of the persons dying from diseases relevant for palliative care died in a hospital, 35.5% in a LTCF, 36.4% at home and 7.7% in other settings, e.g. hospices, or unknown settings (Fig. 1). Place of death varied widely between people with different causes of death. People who died from a liver disease died in a hospital most often (51.4%), followed by people who died from a chronic respiratory disease (35.9%) or stroke (35.2%). People with dementia died in a hospital least often (1.8%), the vast majority of them (85.6%) died in a LTCF. People with cancer died at home most often (54.2%), followed by people who died from chronic heart disease (35.4%) or reno-vascular disease (32.8%).

### Hospital care utilization at the end of life

In the last year of life, 60.2% of the population relevant for palliative care was admitted to a hospital at least once, of which the majority were acute hospital admissions (Table 4). Almost half of the population (47.8%) visited the ED in their last year of life. Admission to an ICU, tube or intravenous feeding and resusciation in hospital occured far less often.

**Table 4:** Percentage of study population (*N* = 109,707) who used different types of hospital care, in various time periods before death^a^

**Type of hospital care**	**Last 360 days**	**Last 180 days**	**Last 90 days**	**Last 30 days**	**Last 7 days**
Hospital admission^a^	60.2%	53.0%	45.5%	32.0%	14.3%
Acute hospital admission^a^	54.4%	47.8%	41.0%	28.8%	13.2%
ED visit	47.8%	41.2%	34.8%	23.9%	10.9%
ICU admission^a^	8.5%	7.1%	6.2%	5.3%	4.4%
Tube or intravenous feeding in hospital	1.6%	1.0%	1.0%	0.7%	0.4%
Resuscitation in hospital	1.0%	0.9%	0.9%	0.9%	0.8%

^a^ Calculations are based on the first day of hospital or *ICU* admissions. It is possible that an admission extends into a time period closer to death

Roughly half of people who received hospital care at any time in the last year of life were found to (also) receive hospital care in the last 30 days of life. In this time period, almost a third of the population (32.0%) were admitted to a hospital, almost a quarter (23.9%) visited an ED and 5.3% of the population were admitted to an ICU.

### Comparing quality indicator scores across 31 healthcare regions

**Fig. 2:**
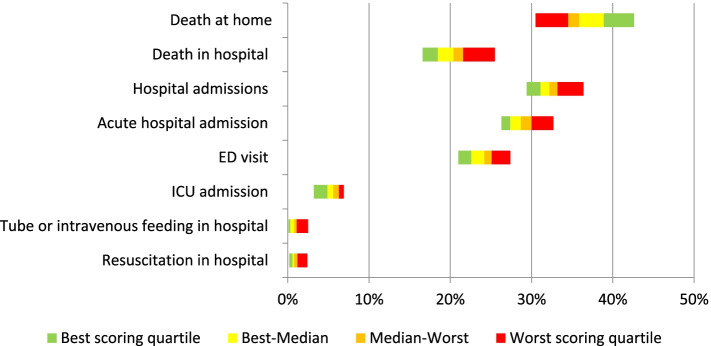
Comparison of indicator scores across 31 healthcare insurance regions (Except for indicators regarding place of death, indicators concern the last 30 days of life)

Variation in quality indicator scores between different healthcare insurance regions in the Netherlands was found for all indicators (Fig. 2), with the largest variation found for the indicators ‘percentage of people dying at home’ (between 30.5% and 42.6%) and ‘percentage of people dying in hospital (between 16.6% and 25.5%). Table 5 shows the scores of the best and worst scoring quartiles per quality indicator. Based on these results, we suggest a best practice performance standard for each indicator, set at the best scoring quartile (Table 5).

**Table 5:** Variation in quality indicator scores and suggestions for best practice performance standards for quality indicators in the Netherlands

**Indicator**^**a**^	**Best scoring quartile**	**Worst scoring quartile**	**Suggested best practice performance standard**
Death at home (%)	42.6–38.9	34.5–30.5	38.9 or higher
Death in hospital (%)	16.6–18.5	21.6–25.5	18.5 or lower
Hospital admission (%)	29.4–31.1	33.2–36.4	31.1 or lower
Acute hospital admission (%)	26.3–27.4	30.0–32.7	27.4 or lower
ED visit (%)	21.0–22.6	25.1–27.4	22.6 or lower
ICU admission (%)	3.2–4.9	6.3–6.9	4.9 or lower
Tube or intravenous feeding in hospital (%)	0.1–0.3	1.1–2.5	0.3 or lower
Resuscitation in hospital (%)	0.2–0.6	1.2–2.4	0.6 or lower

^a^ Except for indicators regarding place of death, indicators concern the last 30 days of life

As can be seen in Fig. 2 and Table 5, the worst scoring quartile of healthcare insurance regions has, in most cases, wider indicator score ranges than other quartiles, indicating that there may be a region that performs far worse than others. However, there is not one region that scores consistently in the best or worst quartile on all eight quality indicators. Analyses show that three regions can be distuingished scoring quite consistenlty in the best quartiles (i.e. on at least five out of eight indicators), and five regions scoring quite consistently in the worst quartiles (data not shown).

## Discussion

By measuring a selection of validated quality indicators for end-of-life care using routinely collected administrative databases, our study provides an insightful overview of the quality of care for Dutch residents who died in 2017 from diseases relevant for palliative care. This is relevant for an international public, as national measurements enable international comparison of health care systems in terms of appropriateness of end-of-life care. Moreover, we were able to develop best practice performance standards based on the comparison of quality indicator scores across 31 healthcare insurance regions in the Netherlands. The method we used is applicable to other countries and settings as well.

### Percentage of people dying at home or in hospital

Our study shows that the preference of the majority of people to die at home [1–4] was in many instances not fulfilled. In 2017, the percentage of people dying at home was 36.4% in the population dying from diseases relevant for palliative care, whereas 20.4% of this population died in hospital. International comparative studies show that the percentage of people dying in hospital is lower in the Netherlands than in many other European countries [17, 18] or Canada [19], consistent with explicit policies in the Netherlands that promote the provision of generalist-led palliative home care [20]. However, we found substantial variation across healthcare insurance regions in quality indicator scores for dying at home and dying in hospital, indicating that there are opportunities for quality improvement within certain regions.

Comparing diseases relevant for palliative care, we found that people with cancer most often died at home. This is not surprising, as many studies point out that cancer patients receive more palliative care and in an earlier stage than non-cancer patients [21–25]. These differences are often attributed to cancer patients’ more predictable illness trajectories [21, 26, 27]. The finding that relatively few people dying from dementia die at home (10.2%) is explained by the fact that these people, when they are in an advanced stage of their disease, often move to a LTCF and die there. Our study showed that only 1.8% of people dying from dementia died in hospital; a percentage that is much lower than what was found in Belgium, where 25.1% of dementia decedents died in hospital in 2015 [13]. This difference is probably due to policy in Dutch LTCFs, where elderly care physicians work who are trained to provide palliative care for nursing home residents until death [28, 29].

### Percentage of people with hospital admissions and treatments

Another finding of our study is that many persons with diseases relevant for palliative care were admitted to hospital (32.0%), visited the ED (23.9%) or were admitted to an ICU (5.3%) in the last month of life. Nevertheless, although the study population was somewhat differently selected, comparing our results to research performed in other countries shows that hospitalizations, ICU admissions and ED visits are far less common in the Netherlands than in Belgium and Canada [13, 19, 30]. As with the indicator scores concerning place of death, the substantial variation between regions in indicator scores concerning hospital admissions and ED visits points to opportunities for quality improvement.

We found little room for quality improvement with regard to the very small percentages of people who received resuscitaion in hospital (0.9%) or tube feeding or intravenoous feeding in hospital (0.7%) in the last month of life. This contrasts with the results of a systematic review of 38 studies, which described the widespread use of non-beneficial treatments at the end of life in hospitals, at least until time of publication of the review in 2016 [31]. Although resuscitation and tube feeding are indicators of inappropriate care at the end of life, even lower percentages or percentages of 0% are not realistic to expect, because life expectancy is sometimes difficult to estimate at the time a treatment decision must be made. In addition, situations may arise in which it is desirable and appropriate for patients and family members to pursue the treatment.

### Towards improvement

Although little variation between healthcare insurance regions was found on the indicators regarding resuscitation and tube or intravenous feeding in hospital, other quality indicator scores regarding place of death and hospital care utilization showed more variation. Thus, as the results show, the type of care patients receive differs between regions. It should be noted that regional varation is not always problematic; variations may exist for good reasons, e.g. differences in patients’ preferences [32]. Variation that does not exist for good reasons (i.e. unwarranted variations) however signals to non-optimal care for patients and can be costly for society [33].

Literature shows that multiple factors may explain variation. For example, distribution of hospital and other care facilities or the local bed supply has been shown to influence care: living near an academic treatment center of more intensive care beds available often leads to more admissions and an extended length of stay in hospital [32].

Our study does not answer the question whether the observed variation between regions is warranted or unwarranted. Nevertheless, according to Westert et al. (2018), a first necessary step to decrease potential unwarranted regional variation is to publicly display findings, which might be uncomfortable for healthcare providers and policymakers. In addition, publishing scores on quality indicators needs to be accompanied by other actions, such as a debate among healthcare providers, policy makers, and patient representatives in which they explore why regional variation exists and attempt to understand its causes and potential remedies. By gaining insight in best practices as performed in regions scoring in the highest quartile, areas for improvement can be pinpointed and subsequently quality improvement interventions can be developed. Research shows that shared-decision making between patients and healthcare providers as well as timely initiation of palliative care integrated into the care for patients at the end of life seems key in improving practice [34–37]. The best practice performance standards as set in this study can be used to measure whether regions perform better in the future. As currently 25% of healthcare insurance regions achieve them, they can serve as ambitious but attainable targets for those regions that currently do not meet these standards.

### Strenghts and limitations

The strength of this study lies in the use of administrative database covering nearly all Dutch residents. Using routinely collected administrative databases and linking them on an individual level has the advantage that healthcare utilization can be reconstructed without needing to collect data through expensive and inherently limited surveys and – especially in light of the challenges of end-of-life care research – individual recruitment and burdening of patients and their family. Such data with national coverage considerably reduce the uncertainty related to sampling errors, selection bias, and recall bias as the data is collected prospectively.

However, the use of administrative databases also has limitations. Quality indicators for administrative databases measure care quality on a population level, but do not necessarily reflect how an individual patient experiences the quality. For example, clinical factors or patient preferences may justify an acute care intervention. Ideally, interpretation of quality indicator scores should therefore be coupled with other measures of quality such as Patient Reported Outcome Measures (PROMs) and patient experience. Another limitation of our study is that we do not know whether we corrected sufficiently for population differences between regions in calculating the variation between regions and the best practice performance standards. Although we corrected for age, gender, cultural background and cause of death, there may be other characteristics (e.g. socioeconomic status, the distribution of care facilities among regions and patient preferences) which influence place of death and hospital care utilization as well, for which we were not able to correct.

## Conclusion

Based on the comparison of quality indicator scores across 31 healthcare insurance regions in the Netherlands, best practice performance standards were derived. Although little variation between healthcare insurance regions was found on the indicators regarding resuscitation and tube or intravenous feeding in hospital, other quality indicator scores regarding place of death and hospital care utilization showed more variation. This regional variation points towards opportunities for quality improvement in care at the end of life. The best practice performance standards as set in this study could serve as attainable targets and facilitate quality improvement.

## Data Availability

The data that support the findings of this study are available from Statistics Netherlands and Dutch Hospital Data but restrictions apply to the availability of these data, which were used under license for the current study, and so are not publicly available. Data are however available from corresponding author upon reasonable request and with permission of Statistics Netherlands and Dutch Hospital Data.

## Abbreviations

COPD: Chronic Obstructive Pulmonary Disease
ED: Emergency Department
HIV: Human Immunodeficiency Virus
ICD-10: International Classification of Diseases
ICU: Intensive Care Unit
LTCF: Long-Term Care Facility
SD: Standard Deviation
SPSS: Statistical Package for the Social Sciences

## References

[1] Gomes B, Higginson IJ, Calanzani N, Cohen J, Deliens L, Daveson BA (2012). Preferences for place of death if faced with advanced cancer: a population survey in England, Flanders, Germany, Italy, the Netherlands. Portugal and Spain Ann Oncol.

[2] Gomes B, Calanzani N, Gysels M, Hall S, Higginson IJ (2013). Heterogeneity and changes in preferences for dying at home: a systematic review. BMC Palliat Care.

[3] Billingham MJ, Billingham SJ (2013). Congruence between preferred and actual place of death according to the presence of malignant or non-malignant disease: a systematic review and meta-analysis. BMJ Support Palliat Care.

[4] Koekoek B (2014). Regie over de plaats van sterven. Een kwantitatieve en kwalitatieve verkenning. (Managing place of death. A quantitative and qualitative exploration).

[5] Dumont S, Jacobs P, Fassbender K, Anderson D, Turcotte V, Harel F (2009). Costs associated with resource utilization during the palliative phase of care: a Canadian perspective. Palliat Med.

[6] De Korte-Verhoef MC, Pasman HR, Schweitzer BP, Francke AL, Onwuteaka-Philipsen BD, Deliens L (2014). Reasons for hospitalisation at the end of life: differences between cancer and non-cancer patients. Support Care Cancer.

[7] De Korte-Verhoef MC, Pasman HR, Schweitzer BP, Francke AL, Onwuteaka-Philipsen BD, Deliens L (2014). General practitioners’ perspectives on the avoidability of hospitalizations at the end of life: A mixed-method study. Palliat Med.

[8] Walsh EG, Wiener JM, Haber S, Bragg A, Freiman M, Ouslander JG (2012). Potentially avoidable hospitalizations of dually eligible Medicare and Medicaid beneficiaries from nursing facility and Home- and Community-Based Services waiver programs. J Am Geriatr Soc.

[9] Gott M, Gardiner C, Ingleton C, Cobb M, Noble B, Bennett MI (2013). What is the extent of potentially avoidable admissions amongst hospital inpatients with palliative care needs?. BMC Palliat Care.

[10] Abel J, Rich A, Griffin T, Purdy S (2009). End-of-life care in hospital: a descriptive study of all inpatient deaths in 1 year. Palliat Med.

[11] De Schreye R, Houttekier D, Deliens L, Cohen J (2017). Developing indicators of appropriate and inappropriate end-of-life care in people with Alzheimer’s disease, cancer or chronic obstructive pulmonary disease for population-level administrative databases: A RAND/UCLA appropriateness study. Palliat Med.

[12] Earle CC, Neville BA, Landrum MB, Souza JM, Weeks JC, Block SD (2005). Evaluating claims-based indicators of the intensity of end-of-life cancer care. Int J Qual Health Care.

[13] De Schreye R, Smets T, Deliens L, Annemans L, Gielen B, Cohen J (2020). Appropriateness of end-of-life care in people dying with sementia: Applying quality indicators on linked administrative databases. J Am Med Dir Assoc.

[14] Etkind SN, Bone AE, Gomes B, Lovell N, Evans CJ, Higginson IJ (2017). How many people will need palliative care in 2040? Past trends, future projections and implications for services. BMC Med.

[15] IKNL/Palliactief. Netherlands Quality Framework for Palliative Care. 2017.

[16] GDPR, recital 27. Available at: Recital 27 - Not applicable to data of deceased persons - GDPR.eu. Accessed 7–1–2022.

[17] Bekelman JE, Halpern SD, Blankart CR, Bynum JP, Cohen J, Fowler R (2016). Comparison of site of death, healthcare utilization, and hospital expenditures for patients dying with cancer in 7 developed countries. JAMA.

[18] Cohen J, Beernaert K, Van den Block L, Morin L, Hunt C, Miccinesi G (2017). Differences in place of death between lung cancer and COPD patients: a 14-country study using death certificate data. NPJ Prim Care Respir Med.

[19] Hill AD, Stukel TA, Fu L, Scales DC, Laupacis A, Rubenfeld GD (2019). Trends in site of death and health care utilization at the end of life: a population-based cohort study. CMAJ Open.

[20] Janssens RJPA, Ten Have HAMJ (2001). The concept of palliative care in the Netherlands. Palliat Med.

[21] Ahmed N, Bestall JC, Ahmedzai SH, Payne SA, Clark D, Noble B (2004). Systematic review of the problems and issues of accessing specialist palliative care by patients, carers and health and social care professionals. Palliat Med.

[22] McKinley RK, Stokes T, Exley C, Field D (2004). Care of people dying with malignant and cardiorespiratory disease in general practice. Br J Gen Pract.

[23] Beernaert K, Cohen J, Deliens L, Devroey D, Vanthomme K, Pardon K (2013). Referral to palliative care in COPD and other chronic diseases: a population-based study. Respir Med.

[24] Pivodic L, Pardon K, Van den Block L, Van Casteren V, Miccinesi G, Donker GA (2013). Palliative care service use in four European countries: a cross-national retrospective study via representative networks of general practitioners. PLoS One..

[25] Evans N, Pasman HRW, Donker GA, Deliens L, Van den Block L, Onwuteaka-Philipsen B (2014). End-of-life care in general practice: a cross-sectional, retrospective survey of ‘cancer’, ‘organ failure’ and ‘old-age/dementia’ patients. Palliat Med.

[26] Exley C, Field D, Jones L, Stokes T (2005). Palliative care in the community for cancer and end-stage cardiorespiratory disease: the views of patients, lay-carers and health care professionals. Palliat Med.

[27] Claessen SJJ, Francke AL, Echteld MA, Schweitzer BPM, Donker GA, Deliens L (2013). GPs’ recognition of death in the foreseeable future and diagnosis of a fatal condition: a national survey. BMC Fam Pract.

[28] Koopmans RTCM, Pellegrom M, Van der Geer ER (2017). The Dutch move beyond the concept of nursing home physician specialists. J Am Med Dir Assoc.

[29] Ten Koppel M, Onwuteaka-Philipsen BD, Van den Block L, Deliens L, Gambassi G, Heymans MW (2019). Palliative care provision in long-term care facilities differs across Europe: Results of a cross-sectional study in six European countries (PACE). Palliat Med.

[30] De Schreye R, Smets T, Annemans L, Deliens L, Gielen B, De Gendt C (2017). Applying quality indicators for administrative databases to evaluate end-of-life care for cancer patients in Belgium. Health Aff (Millwood).

[31] Cardona-Morrell M, Kim J, Turner RM, Anstey M, Mitchell IA, Hillman K (2016). Non-beneficial treatments in hospital at the end of life: a systematic review on extent of the problem. Int J Qual Health Care.

[32] De Man Y, Groenewoud S, Oosterveld-Vlug MG, Brom L, Onwuteaka-Philipsen BD, Westert GP (2020). Regional variation in hospital care at the EOL of Dutch patients with lung cancer exists and is not correlated with primary and long-term care. Int J Qual Healthcare.

[33] Wennberg JE. Tracking medicine: a researcher’s quest to understand health care. Oxford University Press; 2010.

[34] Westert GP, Groenewoud S, Wennberg JE, Gerard C, DaSilva P, Atsma F (2018). Medical practice variation: public reporting a first necessary step to spark change. Int J Qual Health Care.

[35] Boddaert MS, Pereira C, Adema J, Vissers KCP, Van der Linden YM, Raijmakers NJH, et al. Inappropriate end-of-life cancer care in a generalist and specialist palliative care model: a nationwide retrospective population-based observational study. BMJ Support Palliat Care. Epub ahead of print 22 December 2020. 10.1136/bmjspcare-2020-002302. 10.1136/bmjspcare-2020-002302 PMC9120402 33355176

[36] Temel JS, Greer JA, Muzikansky A, Gallagher E (2010). Early palliative care for patients with metastatic non–small-cell lung cancer. N Engl J Med.

[37] Silveira MJ, Kim SY, Langa KM. Advance directives and outcomes of surrogate decision making before death. N Engl J Med. 2010;362:1211–8. 10.1056/NEJMsa0907901 PMC2880881 20357283

[38] Oosterveld-Vlug M, Donker G, Atsma F, Brom L, De Man Y, Groenewoud S (2018). How do treatment aims in the last phase of life relate to hospitalizations and hospital mortality? A mortality follow-back study of Dutch patients with five types of cancer. Support Care Cancer.

